# Preliminary study on the diagnostic value of LEAP-2 and CK18 in biopsy-proven MAFLD

**DOI:** 10.1186/s12876-024-03258-z

**Published:** 2024-05-22

**Authors:** Zhi Liu, Qiao Ren, Hongying Mu, Yuping Zeng, Zhenmei An, He He

**Affiliations:** 1 Department of Endocrinology and Metabolism, West China Hospital, Sichuan University, Chengdu, Sichuan 610041 China; 2 Department of Laboratory Medicine, West China Hospital, Sichuan University, Chengdu, Sichuan 610041 China

**Keywords:** CK18, M30, M65, LEAP-2, MAFLD

## Abstract

Metabolic Dysfunction-Associated Fatty Liver Disease (MAFLD) has become the leading cause of chronic liver disease. Liver biopsy, as the diagnostic gold standard, is invasive and has sampling bias, making it particularly important to search for sensitive and specific biomarkers for diagnosis. Cytokeratin 18 (CK18) M30 and M65 are products of liver cell apoptosis and necrosis, respectively, and liver-expressed antimicrobial peptide 2 (LEAP-2) is a related indicator of glucose and lipid metabolism. Correlation studies have found that all three indicators positively correlate with the liver enzymes alanine aminotransferase (ALT) and aspartate aminotransferase (AST). Through comparison of diagnostic values, it was found that CK18 M65 can better distinguish between healthy individuals and MAFLD; LEAP-2 can effectively distinguish MAFLD from other liver diseases, especially ALD.

**Supplementary Information:**

The online version contains supplementary material available at 10.1186/s12876-024-03258-z.

## Highlights

CK18 M30, M65, and LEAP-2 positively correlate with the liver enzymes ALT and AST.

CK18 M65 is recommended for distinguishing MAFLD patients from healthy controls.

LEAP-2 is recommended for distinguishing other liver diseases from MAFLD.

**Supplementary Information:**

The online version contains supplementary material available at 10.1186/s12876-024-03258-z.

## Introduction

To avoid the stigmatization of “alcohol” in disease nomenclature and promote understanding of the etiology of the disease, a renaming of nonalcoholic fatty liver disease (NAFLD) was proposed in 2020 and 2023 respectively, introducing the term “metabolic”, reflecting scholars’ attention to metabolic factors leading to the fatty liver. The diagnostic criteria have also changed from exclusivity to positivity and NAFLD can coexist with other liver diseases. MAFLD and Metabolic Dysfunction-Associated Steatotic Liver Disease (MASLD) are two kinds of names for NAFLD. The main differences in these three naming conventions are as follows: 1). Naming, both MAFLD, and MASLD avoid the term “alcohol”, and compared to MAFLD, MASLD also avoids stigmatizing the term “fatty liver”; 2) Diagnosis classification: MAFLD cancels the diagnostic classification descriptions for nonalcoholic fatty liver (NAFL) and nonalcoholic steatohepatitis (NASH), while MASLD adds a new definition of MetALD, which refer to patients with liver steatosis who have both alcohol use disorders and metabolic abnormalities; 3) diagnostic criteria: based on liver steatosis, NAFLD needs to exclude alcohol, virus, autoimmune and other causes. MAFLD needs to combine overweight or obesity, type 2 diabetes or metabolic dysfunction. MASLD needs to incorporate one cardiovascular metabolic abnormality (eliminating the requirements for insulin resistance and high sensitivity CRP), and a new diagnostic standard for MASLD in children has been established. Based on different diagnostic criteria, there are also certain differences in the diagnostic population, but the MAFLD, MASLD, and NAFLD populations all have a high overlap rate [1, 2]. According to the data from the Third National Health and Nutrition Examination Survey in the United States, changing the name of NAFLD has no marked effect on its prevalence [3, 4]. Due to the later renaming of MASLD, our study is based on the diagnostic criteria of MAFLD. With the increase in the number of people suffering from metabolic disorders such as obesity and diabetes, the prevalence of MAFLD will gradually increase, which is essentially due to the unhealthy lifestyle of people who eat high-energy diets and lack exercise [5, 6]. MAFLD needs to be paid enough attention to whether it progresses to cirrhosis, liver cancer, liver failure, or an increased risk of death from cardiovascular disease [7]. Unfortunately, liver biopsy remains the gold standard for evaluating fatty liver instead of imaging diagnosis despite of not being sensitive enough. Finding sensitive and specific hematological indexes and establishing a better MAFLD diagnostic model are necessary.

CK18, a type-I intermediate filament protein, is expressed in single-layer epithelial tissues (i.e., liver, pancreas, and intestine). CK18 and CK8 account for 5% of the total cellular protein and bind to form heterodimers, forming the cytoskeleton [8]. CK18 M30 and M65 are fragments of different lengths produced by enzymatic hydrolysis during cell apoptosis and necrosis. Due to the high concentration in hepatocytes, the expression levels of CK18 have been found to increase in various liver diseases (such as MAFLD, drug-induced liver injury, alcoholic-associated liver disease, viral hepatitis, and even liver cancer) [9–12]. A meta-analysis included 25 studies indicate that CK18 M30 and M65 have similar diagnostic abilities for NASH and simple steatosis, with an Area Under the Receiver Operating Characteristic Curve (AUROC) of around 0.8 for diagnosing NASH in NAFLD [13]. While some studies show diagnostic specificity and sensitivity of CK18 in predicting NASH are poor. Therefore, these studies have separately set cutoff values for high sensitivity or specificity or improve diagnostic efficiency by combining other diagnostic indicators. As a result, its drawbacks are also evident: the tangent values cannot be unified; different indicators have differences when combined [14].

LEAP-2, a highly conserved peptide discovered in 2003, has two pairs of disulfide bonds (different from the four pairs of disulfide bonds possessed by LEAP-1). Mainly expressed in the liver and partially removed in the kidneys, the sequence of LEAP-2 is highly conserved in mammals [15]. In addition to its antibacterial function, LEAP-2 has been found to antagonize the effect of ghrelin on growth hormone secretagogue receptor (GHSR), thereby inhibiting the release of growth hormone (GH), reducing food intake and weight loss, and participating in glucose and lipid metabolism. LEAP-2 is associated with glucose and lipid metabolism indicators. In addition to metabolic diseases, it has also been found to be elevated in autoimmune diseases such as rheumatoid arthritis and is associated with inflammatory markers. Compared to healthy controls, the LEAP-2/Ghrelin ratio increases in patients with growth hormone deficiency [16]. Studies have also found that LEAP-2 is highly expressed in girls, especially those in the developmental stage (compared to males and pre-developmental females) [17].

We aimed to investigate the differential expression levels of CK18 M30/M65 and LEAP-2 in MAFLD, healthy controls, and other liver diseases and to evaluate their role in diagnosing MAFLD.

## Methods

**Fig. 1:**
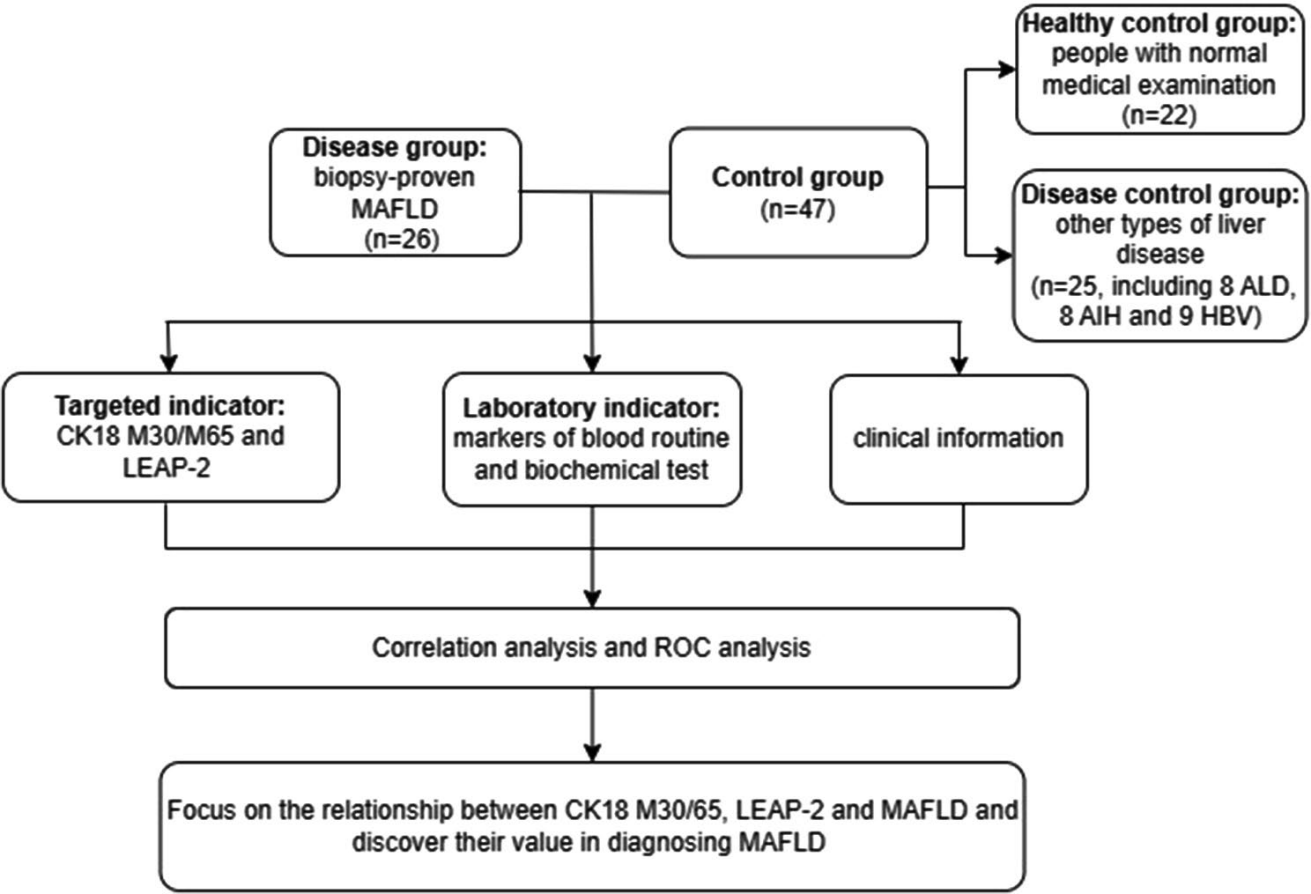
Research flowchart

This study included 26 MAFLD patients, 25 disease controls (8 cases of autoimmune liver disease (AIH), 8 cases of alcoholic liver disease (ALD), and 9 cases of viral hepatitis), and 22 healthy controls who visited West China Hospital of Sichuan University from April to November 2022. The diagnosis of MAFLD is based on histological evidence of liver steatosis combined with metabolic abnormalities (overweight or diabetes or metabolic dysfunction). We collected their clinical information (age, sex, BMI, history of drinking and smoking, current and past medical history) and laboratory indicator results. Nonparametric tests were used for pairwise comparisons between groups. For laboratory indicators, in addition to detecting LEAP-2 and CK18 M30/M63, we chose to include biochemical indicators for routine screening (liver and kidney function tests: ALT, AST, total bilirubin (TBIL), indirect bilirubin (IBIL), direct bilirubin (DBIL), total protein (TP), albumin (ALB), globulin (GLB), alkaline phosphatase (ALP), glutamyl transpeptidase (GGT), uric acid (UA), UREA, CREA, cystatin C (Cys-C), estimated glomerular filtration rate (eGFR); glucose and lipid metabolism tests: glucose (GLU), total cholesterol (CHOL), triglycerides (TG), high density lipoprotein-cholesterol (HDL-C), low density lipoprotein-cholesterol (LDL-C); serum enzyme tests: creatine kinase (CK), lactate dehydrogenase (LDH), hydroxybutyrate dehydrogenase (HBDH)) and blood routine indicators (red blood cell (RBC), hemoglobin (HGB), hematocrit (HCT), mean corpuscular volume (MCV), mean corpuscular hemoglobin (MCH), mean corpuscular hemoglobin (MCHC), platelet (PLT), white blood cell count (WBC), neutrophil (NEUT), lymphocyte (LYMPH), monocyte (MONO), basophils (BASO) and eosinophils (EO). Next, rank correlation analysis was used to investigate the correlation between LEAP-2 and CK18 M30/M65 with routine screening indicators. ROC analysis was performed to compare the diagnostic value of biomarkers for MAFLD. The research flowchart is shown in Fig. 1. The diagnostic criteria of high blood pressure (HBP) are three nondaily measurements of blood pressure systolic blood pressure (SBP) ≥ 140 mmHg and/or diastolic blood pressure (DBP) ≥ 90 mmHg without the use of antihypertensive drugs, as well as a patient’s previous history of hypertension; the diagnosis of diabetes mellitus (DM) is mainly based on the patient’s clinical symptoms, random blood glucose, fasting plasma glucose, oral glucose tolerance test or glycosylated hemoglobin results the diagnostic standard of hyperuricemia (HUA) is that the blood uric acid level on different days exceeds 420 µmol/L; hyperlipidemia (HPL) is characterized by one of the following criteria: CHOL ≥ 6.2 mmol/L, LDL-C ≥ 4.1, HDL-C ≤ 1, or TG ≥ 2.3 mmol/L. Patients with ALD have an alcohol consumption of ≥ 40 g/d in males and ≥ 20 g/d in females or an ethanol content > 80 g/d within two weeks. Viral hepatitis must have evidence of hepatitis virus infection. AIH is mainly based on clinical manifestations, laboratory tests, liver histological characteristics, and accurate clinical diagnosis after excluding other liver diseases.

## Results

The study found that BMI, GLU, UA, WBC and HBP incidence in MAFLD patients were higher and HDL-C expression was lower than those in healthy controls and disease controls. The incidences of metabolic-related diseases such as DM, HUA and HPL and the levels of CK18 M65, ALT, AST, TG, HBDH, GGT and LDH in the MAFLD group were significantly higher than those in the healthy control group. Patients in the MAFLD group were younger than those in the disease control group, and they had fewer drinkers, lower levels of LEAP-2, UREA, Cys-C, ALP, and MONO (%) and higher levels of PLT and NUET (%). The results are shown in Table 1.

**Table 1:** Characteristics of the non-MAFLD and MAFLD groups

Variable	Healthy control group	MAFLD	Disease control group
n	22	26	25
M30 (U/L)	100.00 [100.00, 100.75]	111.50 [100.00, 219.25]	149.00 [100.00, 171.50]
M65 (U/L)	208.50 [157.50, 323.00]*	378.00 [285.50, 622.00]	447.00 [278.50, 840.00]
LEAP-2(ng/mL)	0.21[0.08, 0.56]	0.42 [0.09, 0.86]	1.56[0.48, 2.48]*
Male	8 (36.4%)	9 (34.6%)	12 (48.0%)
Age (years)	28.00 [25.75, 36.75]	29.00 [25.75, 42.00]	50.00 [33.50, 57.50]*
BMI (kg/m^2^)	21.23 [20.07, 24.47]*	37.86 [34.42, 42.72]	22.25 [20.88, 24.29]*
smoker	1 (4.5%)	3 (11.5%)	3 (12.0%)
drinker	0 (0.0%)	3 (11.5%)	10 (40.0%)*
HBP	0 (0.0%)*	23 (88.5%)	2 (8.0%)*
DM	0 (0.0%)*	8 (30.8%)	2 (8.0%)
HUA	1 (4.5%)*	10 (38.5%)	6 (24.0%)
HPL	0 (0.0%)*	11 (42.3%)	10 (40.0%)
TBIL(µmol/L)	10.50 [8.60, 12.45]	11.85 [8.98, 15.70]	15.20 [10.40, 28.15]
DBIL(µmol/L)	3.35 [2.65, 3.83]	3.55 [3.03, 4.15]	4.70 [3.30, 9.75]
IBIL(µmol/L)	7.35 [5.85, 8.78]	8.15 [6.05, 11.80]	9.00 [6.25, 12.90]
ALT (IU/L)	13.00 [10.75, 23.50]*	36.50 [24.75, 70.50]	42.00 [23.00, 98.00]
AST (IU/L)	18.00 [14.75, 19.00]*	25.00 [19.00, 50.75]	39.00 [24.00, 51.50]
TP (g/L)	73.10 [71.95, 74.63]	73.85 [69.40, 78.80]	75.80 [71.20, 78.75]
ALB (g/L)	47.95 [46.80, 49.50]	47.15 [42.85, 48.73]	46.20 [41.95, 49.50]
GLB (g/L)	24.50 [23.58, 27.08]	26.25 [24.68, 30.00]	29.00 [21.65, 33.70]
GLU(mmol/L)	4.57 [4.35, 4.76]*	5.89 [5.27, 7.08]	5.14 [4.61, 5.53]*
UREA(mmol/L)	4.35 [3.68, 4.95]	3.80 [3.08, 4.53]	4.80 [4.20, 6.10]*
CREA(mmol/L)	67.50 [56.00, 74.25]	63.00 [54.00, 73.25]	62.00 [51.50, 78.50]
eGFR(ml/min/1.73 m³)	113.20 [105.32, 123.43]	118.22 [103.16, 125.31]	104.22 [94.06, 116.79]
Cys-C(mg/L)	0.83 [0.78, 0.85]	0.85 [0.75, 1.04]	0.96 [0.89, 1.18]*
UA(µmol/L)	287.50 [262.25, 312.00]*	396.50 [314.50, 479.25]	316.00 [240.50, 430.50]*
TG(mmol/L)	0.82 [0.63, 1.33]*	1.63 [1.17, 2.70]	1.35 [0.92, 1.73]
CHOL(mmol/L)	4.50 [4.07, 4.86]	4.68 [3.82, 5.37]	4.45 [3.74, 5.43]
HDL-C(mmol/L)	1.61 [1.28, 1.77]*	1.10 [0.93, 1.23]	1.31 [1.03, 1.74]*
non-HDL-c(mmol/L)	2.84 [2.40, 3.03]	3.57 [2.73, 4.33]	2.81 [2.27, 3.51]
LDL-C(mmol/L)	2.55 [2.23, 2.71]	2.86 [2.12, 3.43]	2.35 [1.89, 2.84]
ALP (IU/L)	67.50 [53.75, 74.00]	81.50 [65.25, 103.75]	104.00 [76.50, 143.00]*
GGT (IU/L)	15.00 [10.00, 17.00]*	52.50 [19.75, 111.00]	47.00 [21.00, 111.00]
CK (IU/L)	87.50 [61.50, 111.75]	98.00 [67.50, 141.25]	89.00 [65.50, 142.50]
LDH (IU/L)	166.00 [154.50, 181.75]*	201.50 [175.25, 242.50]	196.00 [166.00, 242.00]
HBDH (IU/L)	123.00 [113.75, 135.25]*	146.00 [126.00, 168.50]	141.00 [125.00, 182.00]
RBC (10^12^/L)	4.60 [4.21, 5.23]	4.78 [4.40, 5.15]	4.49 [3.84, 4.87]
HGB(g/L)	138.00 [130.75, 155.50]	136.50 [121.75, 154.50]	135.00 [108.00, 146.00]
HCT	0.43 [0.41, 0.47]	0.44 [0.39, 0.48]	0.42 [0.35, 0.46]
MCV(fL)	93.30 [90.10, 96.25]	91.40 [89.53, 93.03]	93.60 [89.30, 97.65]
MCH(PG)	30.80 [29.25, 31.33]	29.75 [29.20, 30.53]	30.50 [28.70, 31.60]
MCHC(g/L)	326.00 [319.75, 337.25]	326.50 [316.50, 333.00]	322.00 [314.00, 331.00]
PLT(10^9^/L)	238.50 [214.50, 260.5]	257.50 [193.00, 299.00]	166.00 [90.50, 216.00]*
WBC(10^9^/L)	5.71 [4.54, 6.50]*	8.52 [6.90, 10.35]	5.48 [4.07, 6.89]*
NEUT(%)	61.00 [56.18, 68.55]	69.70 [58.03, 83.68]	57.40 [47.35,69.45]*
LYMPH(%)	29.70 [24.60, 35.88]	23.95 [12.03, 32.53]	32.20 [21.40, 38.60]
MONO(%)	5.80 [5.13, 7.23]	5.95 [3.68, 7.23]	7.80 [5.60, 8.65]*
EO(%)	1.05 [0.78, 1.60]	1.25 [0.28, 2.18]	1.30 [0.70, 2.95]
BASO(%)	0.55 [0.38, 0.70]	0.40 [0.30, 0.63]	0.50 [0.30, 0.80]

Data are shown as the median (interquartile range); “*” indicates that the data of this group are significantly different from those of the MAFLD group

**Fig. 2:**
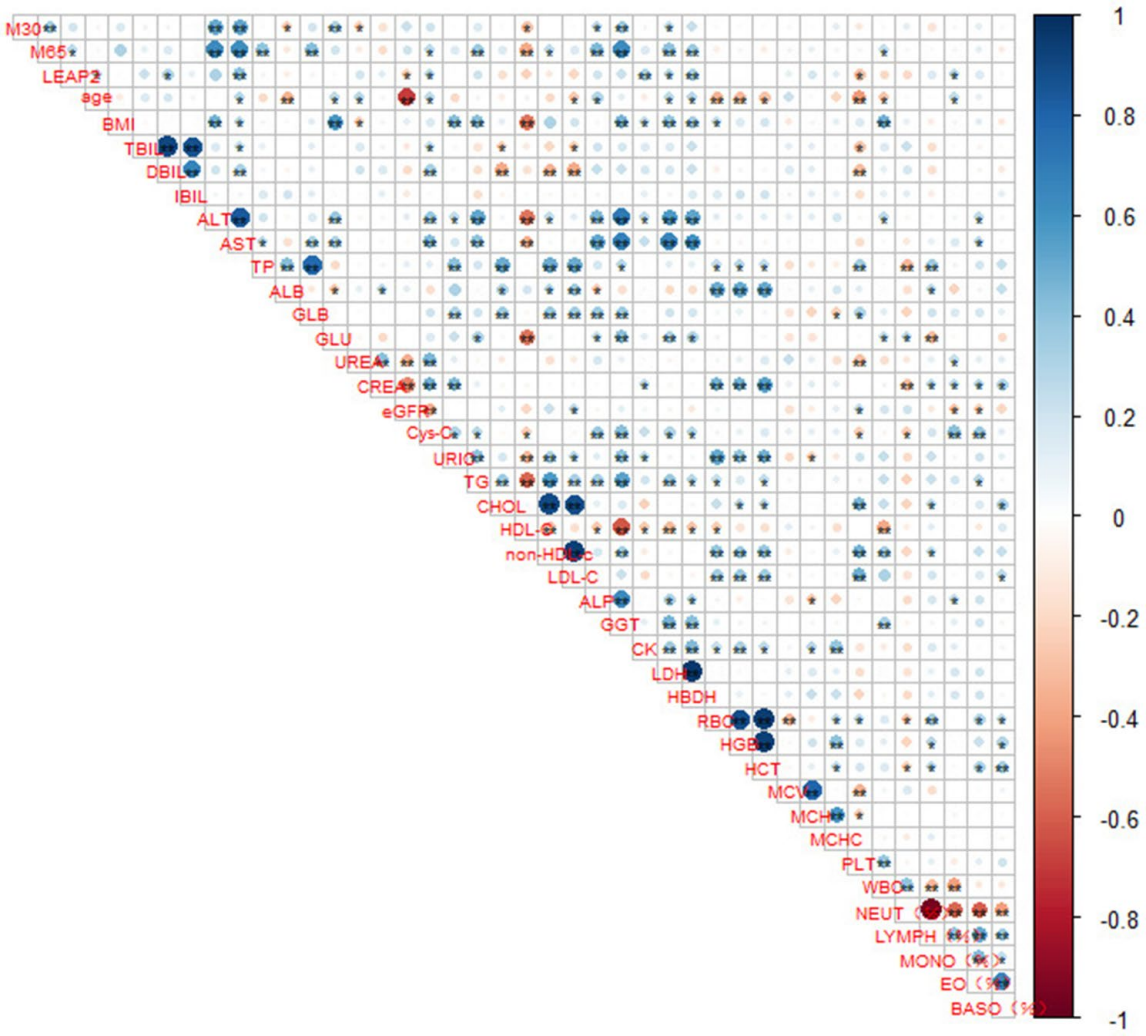
Visualization of the correlation matrix of detection indicators

As shown in Fig. 2, rank correlation analysis of detection indicators revealed that CK18 M30 was positively correlated with ALT and AST, while M65 was mainly associated with ALT, AST, and GGT. In addition, LEAP-2 was weakly positively correlated with ALT and AST.

**Fig. 3:**
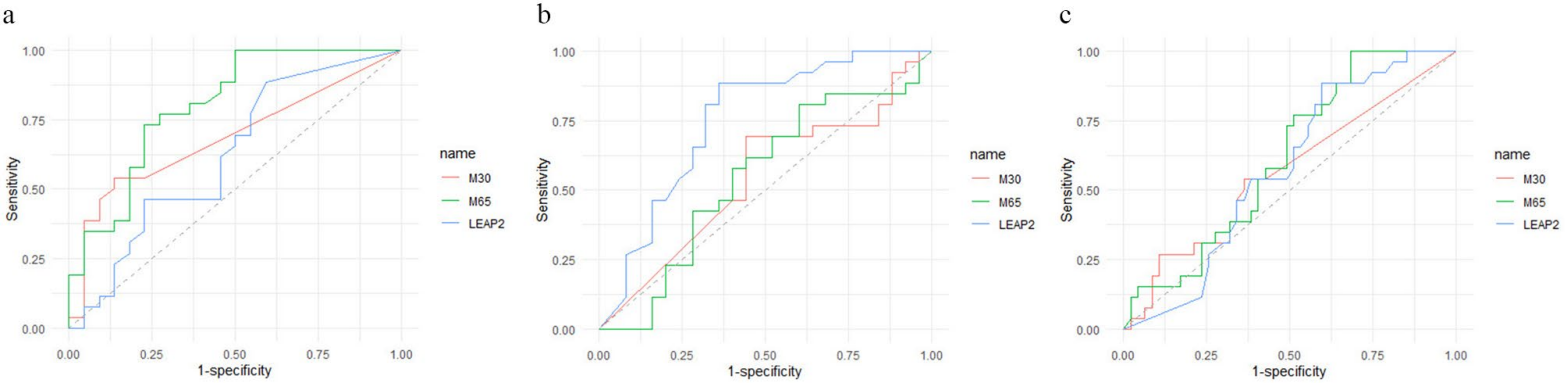
ROC curves for diagnosing MAFLD in different groups: (**a**). the MAFLD group and the healthy control group; (**b**). the MAFLD group and the disease control group; (**c**). the MAFLD group and the control groups (including disease controls and healthy controls)

To discover the value of CK18 M30, M65, and LEAP-2 in diagnosing MAFLD, ROC Curve analyses were performed (Fig. 3). Considering the healthy control group and the disease control group as a control group, AUC for diagnosing MAFLD of M30, M65, and LEAP-2 were 0.563 (95% CI 0.424–0.703), 0.617 (95% CI 0.489–0.745), and 0.412 (95% CI 0.282–0.542), respectively. The three biomarkers’ AUCs for diagnosing MAFLD in healthy controls and MAFLD patients were 0.684 (95% CI 0.532–0.837), 0.802 (95% CI 0.675–0.929), and 0.602 (95% CI 0.439–0.766), respectively. When the cutoff value of M65 was 320.5, its diagnostic sensitivity was 73.1% and specificity was 77.3%. The AUCs of the three biomarkers for predicting MAFLD in disease control and MAFLD were 0.543 (95% CI 0.382–0.704), 0.545 (95% CI 0.382–0.709), and 0.756 (95% CI 0.620–0.892), respectively.

To better determine whether there is a difference in LEAP-2 expression levels between MAFLD and a specific type of other liver diseases, we conducted a supplementary experiment that included patients who visited West China Hospital of Sichuan University from June 2023 to February 2024, consisting of 14 MAFLD, 13 AIH, 12 ALD, and 11 viral hepatitis. The basic information of the patients could be found in the Supplementary Table, which showed that MAFLD patients had higher levels of BMI and UA, and lower levels of DBIL and AST than those of the other three liver diseases. Furthermore, we conducted a ROC analysis and found that the AUROC of LEAP-2 for diagnosing MAFLD in disease control and MAFLD was 0.788 (95% CI 0.650–0.926). The results were shown in Supplementary Fig. 1. For LEAP-2 expression, there was a statistically significant difference between MAFLD and ALD (*p* = 0.000). MAFLD had a lower LEAP-2 than ALD shown in Supplementary Fig. 2. When comparing patient information between the two groups, it revealed that MAFLD had a younger age, fewer drinkers, higher levels of ALB, RBC, HGB, HCT, TG, CHOL, non-HDL-C, LDL-C, and PLT, and lower levels of GLB, MONO%, TBIL and IBIL than ALD.

In the supplementary experiment, drinkers showed an increase in LEAP-2 levels compared to non-drinkers (alcohol consumption 0 g/d) and the difference was statistically significant (*P* = 0.049 < 0.05), shown in Supplementary Fig. 3. Meanwhile, alcohol consumption had a promoting effect on the progression of liver cirrhosis, with an OR value of 12.467 (*P* = 0.000). However, The impact of smoking on the progression of liver cirrhosis has not been found in this study, with an OR value of 3.429 and *P* = 0.078. LEAP-2 was positively related to the liver fibrosis index Fibrosis 4 Score Abbreviated as FIB-4 (R^2^ = 0.2011, *P* = 0.001), and its correlation scatter plot was shown in Supplementary Fig. 4.

## Discussion

The incidence of MAFLD can reach up to 1/4, affecting human health and the economy. The preliminary screening and differential diagnosis of MAFLD are of great significance. CK18 M30/M65, as indicators of liver injury, and LEAP-2, a related indicator of glucolipid metabolism, have received attention. These three indicators are positively correlated with the liver enzymes ALT/AST. When distinguishing between healthy controls, other liver diseases, and MAFLD, the effects of CK18 M30/M65 and LEAP-2 are not satisfactory; when distinguishing between healthy controls and MAFLD, M65 performed better; when distinguishing between other liver disease controls and MAFLD, LEAP-2 performs better.

CK18 has two digestion sites for caspase (Asp238 and Asp396). When apoptosis occurs, cells enter a programmed death process, and fragments of CK18 are released due to caspase digestion. If cells undergo necrosis, it can increase the release of the entire length of CK18 into the bloodstream before it can be enzymatically hydrolyzed [18]. Using a monoclonal antibody, M30 detects a neoepitope of CK18 cleaved by caspase-3 aimed at Asp396 during cell apoptosis. Unlike M30, M65 recognizes both full-length and caspase-cleaved CK18 fragments released during cell necrosis and apoptosis through two monoclonal antibodies [19, 20]. In addition to being related to liver disease, CK18 is associated with some cardiovascular diseases and epithelial tumors. Interestingly, it can indicate MAFLD combined with cardiovascular diseases [19].

As markers of liver injury, both CK18 M30 and M65 are associated with ALT and AST. The same is true in this study [19, 21, 22]. In distinguishing between healthy individuals and MAFLD populations, M30 has a higher specificity at a cutoff value of 108 than M65 at a cutoff value of 320.5 (0.538 vs. 0.731) but a lower sensitivity (0.864 vs. 0.773). However, both have poor efficacy in separating MAFLD from other liver diseases. The most valuable aspect of CK18 lies in differentiating NASH and NAFL, which has been included in the guidelines. Compared to M30, M65 distinguishes fibrosis better, although limited research literature is available [23]. The advantage of CK18 as a biomarker is its stable presence and high specificity for indicating apoptosis [18].

The precursor of LEAP-2 has 77 amino acids. As a cationic peptide, the most prominent native LEAP-2 form with 40 amino acid residues has specific antibacterial activity against gram-positive bacteria and yeast when released into the bloodstream [15]. However, its antibacterial concentration is much higher than the physiological concentration, and another N-terminal cleaved form of LEAP-2 has no antibacterial activity but exists in the blood circulation, indicating that LEAP-2 has other physiological functions worth studying [24]. In addition to liver cells, LEAP-2 is enriched and expressed in intestinal epithelial cells, suggesting that it may be involved in nutrient transport and regulation [24]. In recent years, LEAP-2 has been found to act as a ligand for the GHSR, serving as a reverse agonist of GHSR, downregulating its activity, and acting as an antagonist of ghrelin, blocking its binding to GHSR. When hungry, the secretion of ghrelin and its binding amount with the GHSR increases, promoting growth hormone secretion, further enhancing appetite, and improving feeding behavior. After consuming food, an increase in LEAP-2 inhibits the activity of GHSR and its binding to ghrelin, suppressing GH release. It is worth mentioning that the inhibitory intensity of GHSR activity by LEAP-2 is similar to that of ghrelin-activating receptors. In conclusion, during fasting, the content of LEAP-2 decreases and ghrelin increases; after eating, the changes between the two are opposite [24, 25]. Elevated levels of LEAP-2 were found in mice and humans with liver steatosis and showed a positive correlation with fasting insulin, Homeostatic Model Assessment of Insulin Resistance, and liver fat content related to glucolipid metabolism [26]. This study found that the expression level of LEAP-2 is higher in liver diseases caused by factors other than metabolism than in MAFLD, especially in ALD. Although numerous studies indicated that individuals with higher BMI and blood glucose were associated with elevated LEAP-2 expression, LEAP-2 levels were higher in ALD than in MAFLD. The possible reasons might be that there was not a significant difference in blood sugar levels between the two, and compared to MAFLD, ALD patients in this study were older and had more underlying diseases, poorer liver function, and a higher incidence of fibrosis and cirrhosis. Silvia Ezquerro’s study suggested that the concentration of LEAP-2 might increase with the progression of liver fibrosis [27]. Long-term and excessive alcohol consumption can promote the occurrence of hepatitis, liver fibrosis, and cirrhosis [28]. Even MAFLD patients who consume moderate alcohol within a safe range have an increased risk of liver disease progression, and may even develop into hepatocellular carcinoma [29]. However, in this study, the number of MAFLD individuals who consumed alcohol was relatively small. As a result, we compared the levels of LEAP-2 and the incidence of liver cirrhosis between drinkers and non-drinkers in the supplementary experiment. We found that LEAP-2 was elevated in the population of drinkers, and alcohol consumption could promote the occurrence of liver cirrhosis. After excluding alcohol consumption factors, LEAP-2 did not affect the occurrence of liver cirrhosis (OR = 4.188, *P* = 0.108), but the expression level of LEAP-2 was positively correlated with the liver fibrosis index FIB-4. It is well known that the higher the FIB-4 value, the higher the likelihood of liver cirrhosis. We suggested expanding the sample size and conducting patient follow-up to investigate further the relationship between LEAP-2 levels and liver fibrosis, and cirrhosis, and exclude the influence of alcohol factors. There have been studies indicating that moderate alcohol intake is beneficial and can reduce the risk of cardiovascular disease. However, some studies have limitations, such as small sample sizes and inadequate control of confounding factors. Therefore, a systematic analysis of the Global Burden of Disease Study 2016 proposed the level of alcohol consumption that minimized health loss is zero [30, 31]. LEAP-2 can antagonize the inhibitory effect of ghrelin on insulin by inhibiting ghrelin’s activation of pancreatic GHSR, which is present in α, β and δ cells, as well as pancreatic peptide cells [32]. The hypoglycemic effect of LEAP-2 depends on GHSR [33]. After knocking down LEAP-2 in mice, the expression of receptors and enzymes related to lipogenic/lipolytic metabolism was affected; as a result, liver steatosis, liver enzymes, and triglyceride content were reduced; simultaneously, proteins connected to the IRS/AKT signaling pathway were phosphorylated, resulting in improved insulin sensitivity. Similarly, our study and Ma’s found no correlation between TG and cholesterol and LEAP-2. At the same time, a weak correlation existed between liver enzymes and LEAP-2 in our study [34]. The low concentration of LEAP-2 detected in this study may be related to its rapid clearance and short half-life in the blood circulation [33]. Diagnostic value analysis showed that LEAP-2 has a higher value in distinguishing MAFLD from other liver disease groups, with an AUROC of 0.756 (95% CI 0.620–0.892).

On the one hand, we have identified a new non-invasive diagnostic biomarker LEAP-2 for MAFLD and explored its differential expression in healthy populations, MAFLD, and other liver diseases; On the other hand, we further confirmed the role of CK18 M65 in distinguishing healthy individuals from MAFLD. In future research, two indicators can be combined with other indicators to establish new diagnostic models to improve clinical diagnostic efficiency.

The limitations of this study are as follows. First, the small sample size leads to weak data persuasiveness. Effective sample collection is complex. The disease group samples we collected were all from patients diagnosed with MAFLD through liver biopsy. Liver biopsy is an invasive procedure, with a few patients undergoing surgery. Second, regarding the diagnostic value of LEAP-2 and CK18 M30/M65 for MAFLD, there is still no unified detection method, object, or reference range. The cutoff point values in this article can only serve as a reference and still need clinical validation. Third, this study does not involve mechanism research and cannot analyze the causal dialectical relationship between LEAP-2, CK18, and MAFLD.

## Conclusions

In conclusion, this research has found that CK18 M30/M65 and LEAP-2 are positively correlated with the liver enzymes ALT and AST to a certain extent and can become potential biomarkers for liver diseases. CK18 M65 is more effective in distinguishing between healthy controls and MAFLD than M30 and can be used as a preliminary screening indicator for MAFLD. LEAP-2 can be used to differentiate MAFLD from other liver diseases especially ALD and plays a vital role in differential diagnosis.

In the future, researchers will focus on two main areas of study. Firstly, we will aim to expand the sample size and provide strong evidence to show the diagnostic efficacy of CK18 and LEAP-2 in MAFLD. Secondly, we will study the expression levels of CK18 and LEAP-2 in MAFLD related liver fibrosis and cirrhosis to investigate the roles and possible mechanisms of these indicators in the occurrence and progression of MAFLD.

### Electronic supplementary material

Below is the link to the electronic supplementary material.

Supplementary Material 1

## Data Availability

The datasets used and analyzed during the current study are available from the corresponding author on reasonable request.

## Abbreviations

MAFLD: Metabolic Dysfunction-Associated Fatty Liver Disease
MASLD: Metabolic Dysfunction-Associated Steatotic Liver Disease
NAFLD: nonalcoholic fatty liver disease
NASH: nonalcoholic steatohepatitis
NAFL: nonalcoholic fatty liver
AIH: autoimmune liver disease
ALD: alcoholic liver disease
AUROC: Area Under the Receiver Operating Characteristic Curve
CK18: cytokeratin 18
LEAP-2: liver-expressed antimicrobial peptide 2
ALT: alanine aminotransferase
AST: aspartate aminotransferase
GHSR: growth hormone secretagogue receptor
GH: growth hormone
TBIL: total bilirubin
IBIL: indirect bilirubin
DBIL: direct bilirubin
TP: total protein
ALB: albumin
GLB: globulin
ALP: alkaline phosphatase
GGT: glutamyl transpeptidase
UA: uric acid
Cys-C: cystatin C
eGFR: estimated glomerular filtration rate
GLU: glucose
CHOL: total cholesterol
TG: triglycerides
HDL-C: high density lipoprotein-cholesterol
LDL-C: low density lipoprotein-cholesterol
CK: creatine kinase
LDH: lactate dehydrogenase
HBDH: hydroxybutyrate dehydrogenase
RBC: red blood cell
HGB: hemoglobin
HCT: hematocrit
MCV: mean corpuscular volume
MCH: mean corpuscular hemoglobin
MCHC: mean corpuscular hemoglobin
PLT: platelet
WBC: white blood cell count
NEUT: neutrophil
LYMPH: lymphocyte
MONO: monocyte
BASO: basophils
EO: eosinophils
SBP: systolic blood pressure
DBP: diastolic blood pressure
HBP: high blood pressure
DM: diabetes mellitus
HUA: hyperuricemia
HPL: hyperlipidemia
FIB-4: Fibrosis 4 Score
