# Supplementary material for: Preliminary study on the diagnostic value of LEAP-2 and CK18 in biopsy-proven MAFLD
Source: BMC Gastroenterol. 2024 May 22;24:182. doi: 10.1186/s12876-024-03258-z (PMC11112914; doi:10.1186/s12876-024-03258-z)
Supplement: Supplementary file 1 — Supplementary Material 1 [file 12876_2024_3258_MOESM1_ESM.docx]

Supplementary Table. Characteristics of the MAFLD and disease control groups

| Variable | MAFLD | AIH | viral hepatitis | ALD |
| --- | --- | --- | --- | --- |
| n | 14 | 13 | 11 | 12 |
| LEAP-2(ng/mL) | 0.2[0.08,0.38] | 0.39[0.27,0.79] | 0.28[0.18,0.41] | 0.89[0.5,1.15]* |
| Male | 8(57.14%) | 4(30.77%) | 9(81.82%) | 10(83.33%) |
| Age (years) | 34[28.5,40.5] | 47[37,54.5]* | 46[35,53] | 54[48,61.75]* |
| BMI (kg/m^2^) | 35.91[33.08,37.64] | 22.86[20.06,23.88]* | 24.16[22.65,26.3]* | 23.29[19.18,24.34]* |
| smoker | 2(14.29%) | 4(30.77%) | 3(27.27%) | 3(25%) |
| drinker | 1(7.14%) | 5(38.46%) | 5(45.45%) | 12(100%)* |
| HBP | 6(42.86%) | 1(7.69%) | 1(9.09%) | 1(8.33%) |
| DM | 10(71.43%) | 1(7.69%)* | 1(9.09%)* | 4(33.33%) |
| HUA | 10(71.43%) | 0(0%)* | 1(9.09%)* | 4(33.33%) |
| HPL | 11(78.57%) | 4(30.77%) | 4(36.36%) | 6(50%) |
| TBIL(μmol/L) | 9.9[7.53,14.6] | 20.2[9.85,35.3] | 24.2[15.1,39.3]* | 38.55[24.13,107.85]* |
| DBIL(μmol/L) | 2.55[1.98,3.2] | 9.1[2.9,14.2]* | 7.4[5.3,21.9]* | 17.9[6.25,88.2]* |
| IBIL(μmol/L) | 7.6[5.5,10.8] | 8.1[7,22.45] | 13.3[10.3,18.2]* | 18.15[10.7,34.83]* |
| ALT (IU/L) | 43[20.25,54.75] | 63[24,243] | 60[30,169] | 23.5[17.5,47.25] |
| AST (IU/L) | 23[18.5,29] | 56[34.5,84.5]* | 56[26,83]* | 45.5[30.5,118.25]* |
| TP (g/L) | 72.1[68.05,75.1] | 67.2[60.75,71.65] | 68.5[65.4,76.5] | 71.9[66,76.75] |
| ALB (g/L) | 45.65[43.9,48.53] | 41.2[36.65,43.95] | 39.3[37,46.2] | 34.75[25.13,39.7]* |
| GLB (g/L) | 26.4[24.15,28.28] | 26.5[25,30.2] | 28[25.7,29.3] | 35.05[30.8,45.2]* |
| GLU(mmol/L) | 5.97[5.27,6.43] | 4.86[4.47,5.2]* | 4.53[4.24,4.94]* | 5.49[5.19,6.88] |
| UREA(mmol/L) | 4.65[3.48,5.65] | 4.3[3.9,4.8] | 3.7[2.9,4.9] | 5.3[4.13,8.25] |
| CREA(mmol/L) | 65.5[57.25,81.5] | 59[48,66.5] | 71[67,82] | 70[56.5,77] |
| eGFR(ml/min/1.73m³) | 114.37[103.42,124.15] | 112.8[94.75,117.43] | 105.75[96.52,111.02] | 99.18[89.93,113.52] |
| UA(μmol/L) | 470[386,547] | 259[226,327]* | 319[210,375]* | 279.5[187,443.5]* |
| TG(mmol/L) | 1.63[1.23,2.42] | 1.55[0.79,2.04] | 0.89[0.59,1.37]* | 0.85[0.68,1.12]* |
| CHOL(mmol/L) | 4.78[4.5,5.75] | 4.53[3.42,5.33] | 3.17[2.14,4.41]* | 3.67[2.31,4.2]* |
| HDL-C(mmol/L) | 1.11[0.95,1.19] | 1.26[1.01,1.86] | 1.14[0.84,1.29] | 1.21[0.44,1.88] |
| non-HDL-C(mmol/L) | 3.76[3.44,4.56] | 3.06[1.86,3.84] | 1.7[1.49,3.34]* | 2.39[1.44,2.86]* |
| LDL-C(mmol/L) | 3.3[3.1,3.72] | 2.42[1.86,3.14] | 1.52[1.33,2.89]* | 1.62[0.95,2.19]* |
| ALP (IU/L) | 71.5[64,85.75] | 197[140,273.5]* | 126[77,197] | 118.5[65,175.25] |
| GGT (IU/L) | 41.5[21,53.25] | 148[60.5,218.5]* | 43[16,145] | 53[42.25,70.75] |
| CK (IU/L) | 83[49,132] | 60[51.5,92.5] | 85[69,118] | 77.5[55.25,131.75] |
| LDH (IU/L) | 200[172,242] | 171[147,198] | 194[174,248] | 203[179,252.5] |
| HBDH (IU/L) | 138[128,171] | 127[114,149] | 148[127,182] | 153.5[149.25,166.25] |
| RBC (10^12^/L) | 5.37[4.79,5.69] | 4.11[3.88,4.51]* | 4.85[4.02,5.58] | 3.84[2.97,4.47]* |
| HGB(g/L) | 143.5[138.5,160.25] | 118[102.5,138.5]* | 143[122,158] | 102.5[90.75,136.5]* |
| HCT | 0.45[0.44,0.49] | 0.39[0.33,0.43]* | 0.44[0.38,0.5] | 0.33[0.29,0.41]* |
| MCV(fL) | 89.8[86.3,92.48] | 92.3[86.25,94.9] | 94.2[88.7,96.1] | 96.65[86.15,100.7] |
| MCH(pg) | 29.05[28.13,29.78] | 28.5[26.6,30.95] | 30.6[30,31.9] | 31.2[27.1,33.45] |
| MCHC(g/L) | 323[315,327.25] | 322[305,326.5] | 336[320,337] | 328.5[306.75,342] |
| PLT(10^9^/L) | 265.5[208.25,328] | 121[81,233] | 132[59,152]* | 57.5[40.75,118.5]* |
| WBC(10^9^/L) | 8.35[6.46,9.85] | 4.73[2.49,6.15]* | 5.89[3.23,7.18]* | 5.56[3.28,8.27] |
| NEUT（％） | 66.1[60.95,69.78] | 58.9[52.1,69.55] | 55.3[51.3,66.8] | 65.65[55.55,70.65] |
| LYMPH（％） | 25.45[23.15,32.33] | 28.6[18.05,34.35] | 32.8[26.3,40.7] | 20.85[18.05,30.68] |
| MONO（％） | 4.95[4.48,6.15] | 8.8[6.4,10.85]* | 6.5[4.3,9.1] | 9.5[5.85,11.38]* |
| EO（％） | 1.25[0.9,1.9] | 3[1.15,4.05] | 2.6[1.1,3.5] | 2.35[1.15,2.93] |
| BASO（％） | 0.5[0.38,0.6] | 0.5[0.25,0.75] | 0.6[0.4,0.8] | 0.6[0.25,0.8] |

Data are shown as the median (interquartile range); “*” indicates that the data of this group are significantly different from those of the MAFLD group.


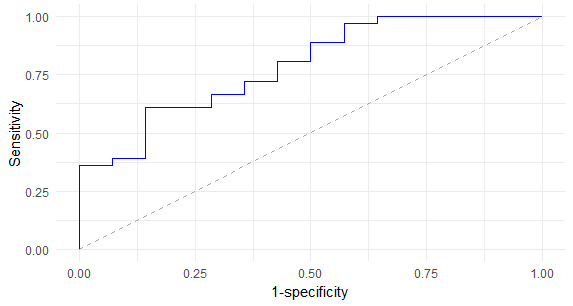


Supplementary Figure 1. ROC curves of LEAP-2 for diagnosing MAFLD in the MAFLD group and the disease control group


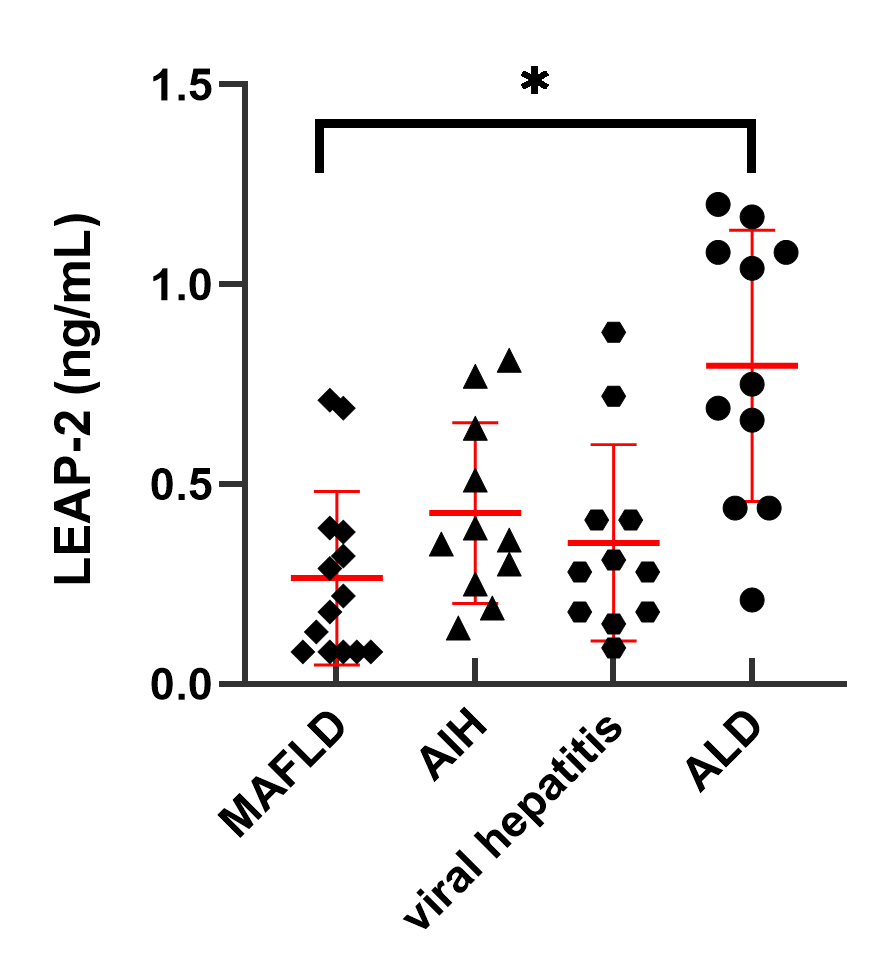


Supplementary Figure 2. Expression of LEAP-2 in MAFLD and other liver diseases;

* p <0.05


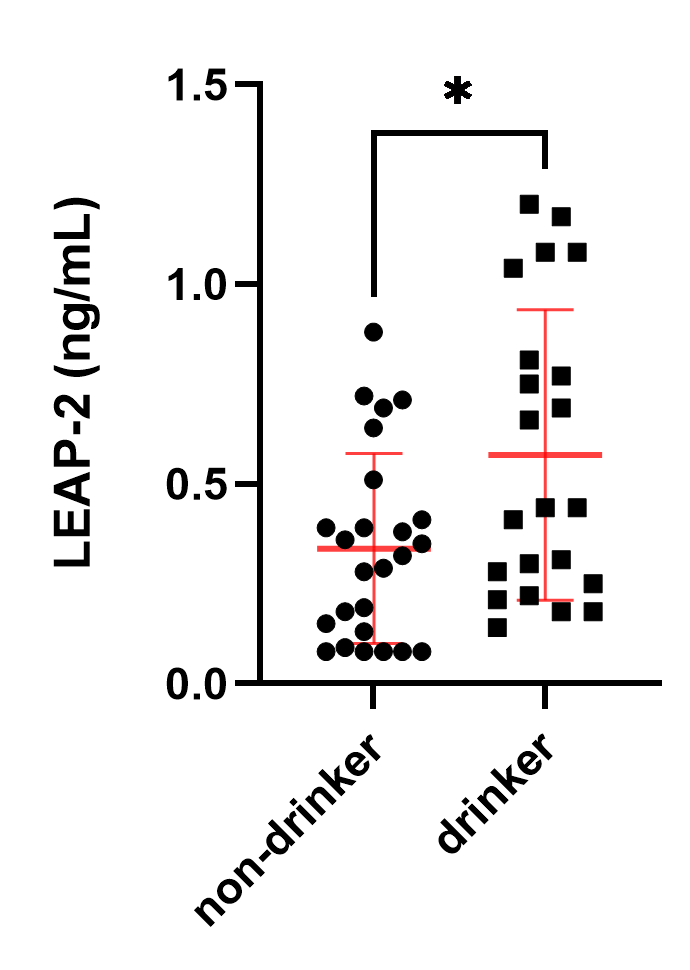


Supplementary Figure 3. Expression of LEAP-2 in non-drinkers and drinkers,

* p <0.05


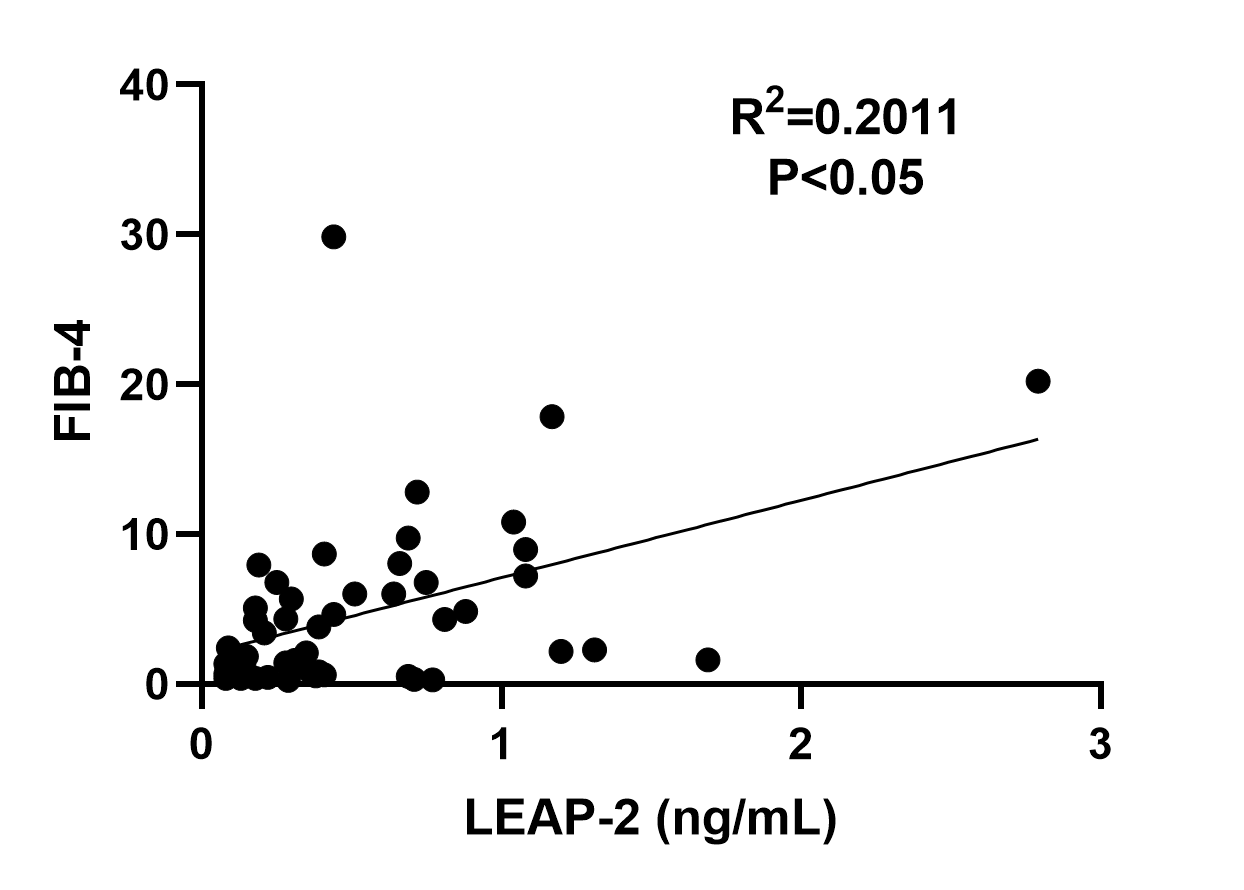


Supplementary Figure 4. Correlation scatter plot of LEAP-2 and FIB-4
